# iCrotoK-PseAAC: Identify lysine crotonylation sites by blending position relative statistical features according to the Chou’s 5-step rule

**DOI:** 10.1371/journal.pone.0223993

**Published:** 2019-11-21

**Authors:** Sharaf Jameel Malebary, Muhammad Safi ur Rehman, Yaser Daanial Khan

**Affiliations:** 1 Department of Information Technology, King Abdul Aziz University, Rabigh, Kingdom of Saudi Arabia; 2 Department of Computer Science, School of Systems and Technology, University of Management and Technology, Lahore, Pakistan; Swiss Institute of Bioinformatics, SWITZERLAND

## Abstract

Among different post-translational modifications (PTMs), one of the most important one is the lysine crotonylation in proteins. Its importance cannot be undermined related to different diseases and essential biological practice. The key step for finding the hidden mechanisms of crotonylation along with their occurrence sites is to completely apprehend the mechanism behind this biological process. In previously reported studies, researchers have used different techniques, like position weighted matrix (PWM), support vector machine (SVM), k nearest neighbors (KNN), and many others. However, the maximum prediction accuracy achieved was not such high. To address this, herein, we propose an improved predictor for lysine crotonylation sites named iCrotoK-PseAAC, in which we have incorporated various position and composition relative features along with statistical moments into PseAAC. The results of self-consistency testing were 100% accurate, while the 10-fold cross validation gave 99.0% accuracy. Based on the validation and comparison of model, it is concluded that the iCrotoK-PseAAC is more accurate than the previously proposed models.

## 1. Introduction

In all the living organism, the cells contain the chromosomes having the stored information, which deals with the normal body functions. Specifically, chromosomes contain DNA (Deoxyribonucleic acid), the polymer of deoxyribonucleotides [1]. Almost 80–90% of the DNA is considered as junk, whose exact functioning is not identified yet; but for the rest, it has been discovered. Although, the complexity of the Genomic sequences within the chromosomes follows highly patterned and efficiently organized methodology, the main function of the DNA is to replicate and encode the body proteins [2]. These body proteins are formed by unwinding of the DNA by semi-conservative methods. Both strands of DNA encode its signals to the Messenger RNA (mRNA) by the process of transcription. It is followed by the translation, in which protein sequence is transmitted to the transfer ribonucleic acid (tRNA). At the end, the tRNA terminates the process by joining amino acids over the ribosome (organelle) and forming protein exactly according to the gene sequence. After this translation, post-translational modification can take place, which can either activate or inactivate the function of that protein [3].

Crotonylation is a reversible post-translational modification process, which usually takes place over lysine [4] residues of a protein. It holds immense importance, with respect to its various effects on the body’s metabolism, genetic expressions and multiple diseases i.e. carcinomas and malignancies etc. [5]. Lysine is an essential amino acid with a basic side chain and a product of putrefaction in the gut. The chemical structure of the lysine contains a carboxylic group, amino group, hydrogen and a basic R group, attached to the central carbon of the amino acid backbone [6].

Up till now, a couple of computational based techniques have been proposed for the prediction of lysine crotonylation sites in proteins. According to a theory that both crotonylated peptides and non-crotonylated peptides are produced by particular mechanisms, Huang and Zeng [7] proposed an automated predictor, named CrotPred, for the prediction of histone crotonylation sites in proteins. Later on, Qiu et al. [8] provided a mechanism for the identification of lysine crotonylation sites, which utilized position weight amino acid composition, to identify CrotoK sites using a support vector machine (SVM) algorithm. Notwithstanding, the prescient accuracies of CrotPred and Qiu's technique achieved just 79.41% and 71.69%, individually. The prediction accuracy of the over two techniques is as yet not much high. It ought to likewise be noticed that the over two techniques [8] did not provide good accuracy results. Later on, Ju et al. proposed CKSAAP_CrotSite to identify lysine crotonylation sites with an accuracy of 98.11% [9] and Qiu et al. proposed iKcr-PseEns to identify lysine crotonylation sites in histone proteins with an accuracy of 94.49% [10].

The efficiency of the previous research processes lacks the relative positioning and composition information which holds immense importance, with two basic goals; to make the relevant theoretical study and to give scientist an easier layout for research purpose. In order to achieve these goals, a 5-steps rule [11–18] should be employed, as used by the researchers in the past [13, 19–22]. These steps include (i) collection of a standardized dataset, (ii) mathematical formulation of features and association with biological target classes, (iii) training of predictor via operational algorithm, (iv) objective evaluation and validation of results, and (v) implementation of webserver for proposed predictor. Although the credibility of the methods has been improved successively still its upper limit could touch the satisfactory value. We, herein, propose a predictor named iCrotoK-PseAAC which aims to identify CrotoK sites with improved efficiency than previously reported methods, using PseAAC and Chou’s 5-steps rule.

## 2. Materials and methods

**Fig 1:**
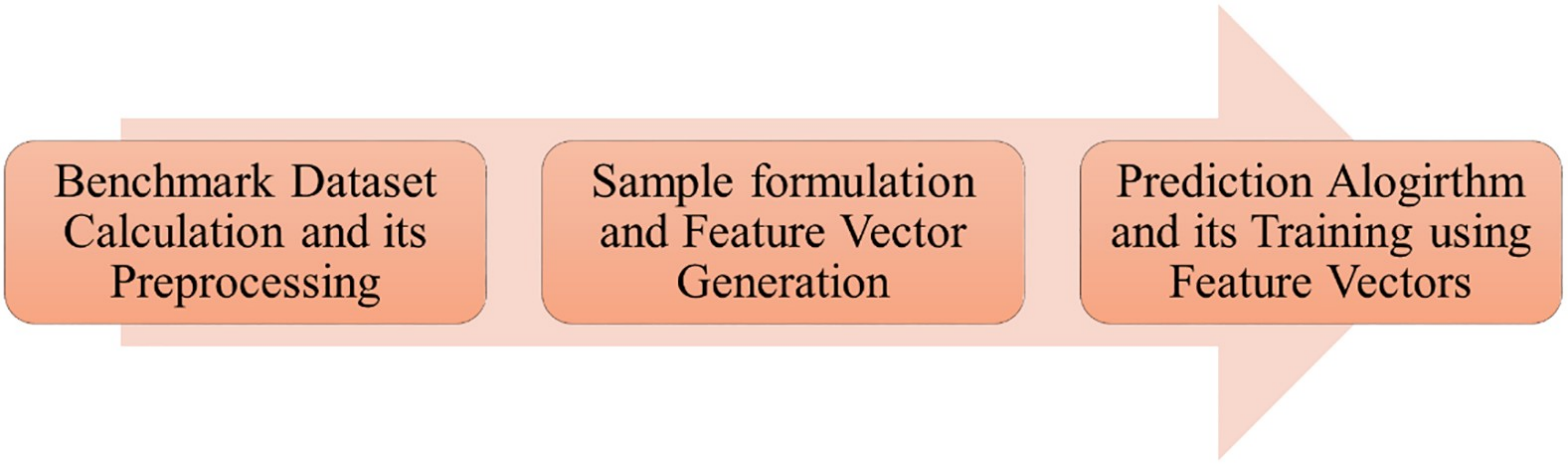
Three steps of methodology.

In the following section, the primary three portions of the 5-steps rule are being discussed. The methodology is being presented in the flowchart in Fig 1.

Using graphic approaches to study biological and medical systems can provide an intuitive vision and useful insights for helping analyze complicated relations therein as shown by the eight master pieces of pioneering papers from the then Chairman of Nobel Prize Committee Sture Forsen [23, 24], and many follow-up papers [25–29]. They are very useful for in-depth investigation into the topic of the current paper, and we will use them in our future efforts.

### 2.1. Benchmark dataset

Chou’s peptide formulation [30] was used for an accurate elaboration of samples, which had generally utilized in computational biology and bioinformatics; for example, in predicting the signal peptide cleavage sites [31], nitrotyrosine sites [32], methylation sites [33], hydroxylysine and hydroxyproline sites [34], lysine ubiquitination sites [35], protein-protein binding sites [36], phosphorylation sites [37, 38], lysine succinylation sites [39], and, numerous lysine PTM sites [40].

Collection of benchmark dataset was performed from Universal Resource of Protein (UniProt). A query was applied to database using advanced search options and the sequences with PTM/Processing annotations were chosen. In modified residue field, ‘crotonyllysine’ phrase for positive samples was used. For negative data, a converse query was used. Thus, by these searches, 2665 positive and 868 negative datasets were filtered out. Redundancy and duplication from dataset was removed by using CD-HIT webserver [41] with a threshold of 0.6. The reduced dataset was used to extract sites from each sequence of positive and negative set, and a subsequence of length 41 was extracted for all positive and negative sites. Experiment was repeated for other lengths, i.e. 21 and 61, however, best results were achieved for 41. Before proceeding to the next step, we have removed the unnecessary information like some of the special characters and notations (B, J, O, U, X and Z) and spaces from the dataset of positive and negative samples of protein. Thus, a total of 378 positive and 500 negative samples were achieved.

Stated by Chou’s scheme [30], the CrotoK site-having peptide sample can be usually defined as

$$P_{\xi }(K)=R_{-\xi }R_{-(\xi -1)}\cdots R_{-2}R_{-1}KR_{+2}\cdots R_{+(\xi -1)}R_{+\xi }$$ (1)

Importance of amino acid K is shown by emphasizing the symbol using double strike, the subscript ξ is a number ranging [-ξ, +ξ] which include both positive and negative numbers excluding 0, R_−ξ_ gives the -ξ-th upstream amino acid between the range, the *R*_+ξ_ the +ξ-th gives the downstream amino acid other than the R_−ξ_, etc. The (2ξ + 1)-tuple peptide sample $P_{\xi }(K)$ can be more arranged in further two classes as shown in Eq (2).

$$P_{\xi }\left(K\right)\in \{\begin{array}{r} \;P_{\xi }^{+}\left(K\right),\;for\;the\;CrotoK\\ P_{\xi }^{-}\left(K\right),\;for\;non-CrotoK \end{array}$$ (2)

Where $P_{\xi }^{+}(K)$ indicate the positive CrotoK sites with K at mid, while $P_{\xi }^{-}(K)$ indicate the non-CrotoK sites, and the mark ∈ means “a member of” in a group of sets.

Data-collection for benchmarks was based on two types of the dataset, one was used for training which we called training dataset and the other dataset was used for testing which we called testing dataset. There is no compelling reason to isolate a benchmark dataset into two datasets if you have used either of jackknife or subsampling using k-fold cross validation (where k = 5 or k = 10) because the results were generated using different folds [13, 21]. In this research, the value of ξ was 20 which was then put in the equation (2ξ+1) which equals to 41 characters in the subsequence using Eq (2). As needs are, the benchmark dataset for this investigation can be accumulated into S as shown in Eq (3).

Where $S^{+}$ have 378 positive samples, $S^{-}$ have 500 negative samples while ∪ is denoting “union” in a group of hypotheses. As per user’s convenience, the 378 + 500 = 878 example sequences are given in S1 File [42].

$$S=S^{+}\;\cup \;S^{-}$$ (3)

### 2.2. Sample formulation

Due to the recent advancement in the biological computation because of the increase in the biological sequence, one of the popular problems is to compute the biological sequences using a discrete model or vector by the keeping the actual sequence intact.

Since all the conventional machine learning algorithms deal with the vectors and not directly with the sequence samples [43], so pseudo amino acid composition or PseAAC [44] was then created to solve such problems. With the creation of PseAAC or Chou’s PseAAC [45, 46], the field of biomedicine and drug development industries started to use it [47, 48] and almost every area of study in the field of computational proteomics [49–51] took interest in it. Due to its rapid and sudden popularity, many open source software [45, 52] were developed to generate the various sequences of the PseAAC. Due to the increasing popularity of the PseAAC, several webservers [53] were created to generate different DNA/RNA sequence and has proven itself to be very useful in the field of computational genomes [54]. Pse-in-One [55] was developed to generate any number of desired feature vectors according to the user’s focuses for both DNA/RNA and protein/peptide sequences.

As indicated by Eq (3) and formulated in PseAAC, the sample sequences of the peptide in S1 File are extracted as suggested in [30]

$$P{}_{ʂ=7}(K)={\left[{Ӑ}_{1}{Ӑ}_{2}\cdots {Ӑ}_{\Omega }\right]}^{T}$$ (4)

Where Ă_1_Ă_2_⋯Ă_Ω_ will be characterized by how to extricate valuable features from samples of a peptide sequence, and T is the transpose administrator.

The UniProt database provides number of ways to extract the required data. During this study the data was extracted by parsing XML files generated by UniProt as a result of query that search for all proteins containing the required modified residue. Different values of Ω were tried in order to find the most optimal one. Extensive trials of experimentation and probing showed that Ω = 20 yielded best results. Hence the benchmark data was formed using 20 residues at either terminals of the crotonylation site. Subsequently, the benchmark dataset contains peptide sequences of length 41 as given in S1 File can be rearranged to the accompanying simpler to deal with frame and their reduction is shown in Eq (5).

$$P=R_{1},R_{2},R_{3}\cdots R_{19},R_{20},R_{21}\cdots R_{39},R_{40},R_{41}$$ (5)

Where R_21_ is the modified residue K and *R*_*i*_ for *(i = 1*, *2*, *… 41; i ≠ 21)* can be any of the 20 native amino acids or the dummy code X as discussed before. For further clarification, 20 native amino acids were given numerical values ranging from 1 to 20 with respect to the alphabetic order of their letter code and remaining 21 dummy amino acids were given the numeric values ranging from 21 to 41.

#### 2.2.1. Site vicinity vector (SVV)

There are various remaining sites which are similar to Post-Translational Modification (PTM) in the chain of polypeptide and there exist many characteristics that channel us towards the modification. Other than the previously discussed characteristics, the other neighboring residues of a site are also important in which PTM is observed [56]. The sequence which contains the potential PTM residue site from the primary sequence of the protein is known as Site Vicinity Vector (SVV).

Following is the primary sequence having the potential PTM site with its surrounding residues and in Eq (6) it is denoted as

$$Ρ=[\mu_{1}\ldots \mu_{x-2},\mu_{x-1},\mu_{x},\mu_{x+1},\mu_{x+2},\ldots ..\mu_{n}]$$ (6)

Sub-sequence of the primary sequence is SVV which is represented as

$$S={[\mu }_{x-k}\ldots \mu_{x-2},\mu_{x-1},\mu_{x},\mu_{x+1},\mu_{x+2},\ldots ..\mu_{x+k}]$$ (7)

After thorough experiments, the result shows that the minimum constant value best suited for k is 20 in our specific scenario. Each characteristic in SVV shows the specific amino acid already known, out of given 20 amino acids in which a unique numerical value are assigned to each amino acid ranging from 1 to 20.

#### 2.2.2. Calculation of statistical moments (SM)

Statistical moment approach is utilized to consider the quantitative explanation for benchmark dataset of protein sample sequence to define the dimensions and components of Eq (6). To keep the crucial information safe regarding the protein samples’ sequence order, as it is necessary that the benchmark dataset of protein samples have the amino acid residues to be in a specific order. Different types of factors were considered while gathering the information of protein samples using different variables such as order of moments in which some of the moments for data evaluation and some for peculiarity of data and some for orientation from enormous amount of gathered protein samples and some of them were elaborated by the mathematicians using distributions and polynomial functions [13, 21, 37, 38, 56–65].

We have calculated Hahn moments by using Hahn polynomial, raw moments by using probability distribution of benchmarked dataset of protein samples and central moments using mean, variance and asymmetry of information and these are computed for iCrotoK -PseAAC predications. Their results are scale variant [63], whereas the central moment is both scale variant and vicinity variant [63, 65] and the results computed in raw moments of mean, variance and asymmetry are then further used in the calculations of the central moment.

Moments of the scale variants are not used, and each method describes the qualified values [66, 67] of the data in 2 dimension (2D) square matrix P (in which rows and columns are same and denoted as m) with dimension *m x m* as written in Eq (8) and accommodate all of the protein samples residues in our proposed methodology.

⌊P11P12⋮Pm1P12…P22…⋮Pm2…P1mP2m⋮Pmm⌋ (8)

Now the generated square matrix ***P*** from Eq (8) is passed to the function *ω* which transformed that ***P*** matrix into ***P’***. This can be mathematically written as *P*′ = *ω*(*P*)) with the 3rd degree of statistical moments (means rows and columns should not exceed 3 from the moments matrix P) and then the newly generated matrix ***P’*** is used by Hahn moment, which speeds up our calculation. The property of ***P’*** being a square matrix made Hahn moment orthogonal in which the inverse propriety was preserved and could be converted back to ***P*** using the inverse function of discrete Hahn moment as shown in Eq (9).

Hnu,z(r,M)=(M+Z-1)n(M-1)n×∑i=0n(-1)i(-n)i(-r)i(2M+u+z-n-1)i(M+z-1)i(M-1)i×1i!¯ (9)

The pochhammer symbol and the gamma operator is applied in (9) are described in [56, 67]. The normalized orthogonal Hahn moments calculation was performed using Eq (10)

$$h_{rs}=\sum_{a=1}^{M-1}\sum_{b=1}^{M-1}\partial_{rs}H_{r}^{\overset{∼}{u,z}}(b,M)H_{s}^{\overset{∼}{u,z}}(a,M),n=0,\;1,\;2,\;3\ldots M-1$$ (10)

Highly imported information is conserved by central moments which is related to the variance, asymmetry and means of the sample proteins and its calculation is carried out using Eq (11).

$$I_{rs}=\sum_{a=1}^{k}\sum_{b=1}^{k}{\left(a-\bar{x}\right)}^{r}{\left(a-\bar{y}\right)}^{s}\partial_{ab}$$ (11)

In the end, benchmark dataset of samples protein’s information is gathered using the calculation of raw moments through distribution probability as shown in Eq (12)

$$M_{rs}=\sum_{a=1}^{k}\sum_{b=1}^{k}a^{r}b^{s}\partial_{ab}$$ (12)

Raw moment’s degree is *r+s* and *M*_00_, *M*_01_, *M*_02_, *M*_10_, *M*_11_, *M*_20_, *M*_12_, *M*_21_, *M*_03_ *and M*_30_ of 3rd degree is mediated here.

#### 2.2.3. Calculations of position relative incidence matrix (PRIM)

The primary sequence plays a vital role in calculating the hidden patterns from the sequence of the protein. The positional information of the protein benchmarked dataset and the residues in it play a key role in central mathematical paradigm of the model and 20x20 matrix is formed by using quantization of relative positional information of the residues in the amino acid and we called it *H*_*PRIM*_.

$$H_{PRIM}=\left[\begin{array}{cccc} H_{1\to 1} & H_{1\to 2}\cdots & H_{1\to j}\cdots & H_{1\to 1}\\ H_{2\to 1} & H_{2\to 2}\cdots & H_{2\to j}\cdots & H_{2\to 20}\\ H_{i\to 1}^{\vdots } & H_{i\to 2}^{\vdots }\cdots & H_{i\to j}^{\vdots }\cdots & H_{i\to 20}^{\vdots }\\ H_{N\to 1}^{\vdots } & H_{N\to 2}^{\vdots }\cdots & H_{N\to j}^{\vdots }\cdots & H_{N\to 20}^{\vdots } \end{array}\right]$$ (13)

In Eq (13) *i* represents the specific row number and *j* represents the specific column which contains the sum of the first appearance of the *i*^*th*^ residue with respect to the *j*^*th*^ relative position and it generates 400 coefficients which then reduced to 30 generated coefficients by using the statistical moments [56].

#### 2.2.4. Calculations of reverse position relative incidence matrix (RPRIM)

The *H*_*RPRIM*_ is calculated using the reverse of protein samples instead of using the actual ones which we have used in *H*_*PRIM*_, so that we can be able to find out the ambiguities in the samples of proteins, the matrix is shown in Eq (14).

$$H_{RPRIM}=\left[\begin{array}{cccc} H_{1\to 1} & H_{1\to 2}\cdots & H_{1\to j}\cdots & H_{1\to 1}\\ H_{2\to 1} & H_{2\to 2}\cdots & H_{2\to j}\cdots & H_{2\to 20}\\ H_{i\to 1}^{\vdots } & H_{i\to 2}^{\vdots }\cdots & H_{i\to j}^{\vdots }\cdots & H_{i\to 20}^{\vdots }\\ H_{N\to 1}^{\vdots } & H_{N\to 2}^{\vdots }\cdots & H_{N\to j}^{\vdots }\cdots & H_{N\to 20}^{\vdots } \end{array}\right]$$ (14)

The *H*_*RPRIM*_ gives the same amount of dataset but generates the different values and generates the same number of coefficients as *H*_*PRIM*_ in Eq (14) does and which can be reduced the same way we reduced *H*_*PRIM*_ [13, 21, 56].

#### 2.2.5. Determination of frequency vector (FV)

The vector containing the frequencies of all the residues of the protein in the benchmark dataset and we called it frequency vector which has the property to sustain the compositional and distributional information regarding the samples of the protein sequence. FV is shown in Eq (15).

$$FV=[f_{1},\;f_{2},f_{3},f_{4},f_{5},..{,f}_{20}]$$ (15)

Where each *f*_*i*_ holds the frequency by the alphabetic order of each residue of amino acid in the sequence.

#### 2.2.6. Determination of accumulative absolute position incidence vector (AAPIV)

The cumulative frequency distribution of the residues of amino acids in protein polypeptide chain related to the composition of the protein is known as accumulative absolute position incidence vector (AAPIV) and it only holds the information of absolute position thus the information regarding the relative position is lost. A vector of 20 elements is then formed (as shown in Eq (18) which formed based on primary structure given in Eq (16) where each element in this vector represents the sum of ordinal values of its occurrences of respective residue at *p*_1_, *p*_2_, …, *p*_*n*_ locations within the primary structure as shown in (17).

$$\alpha_{p1}^{i},\;\ldots ,\;\alpha_{p2}^{i},\;\ldots ,\;\alpha_{pn}^{i}$$ (16)

$$\mu_{i}=\sum_{k=1}^{n}p_{k}$$ (17)

$$AAPIV=\{\mu_{1}.\mu_{2},\ldots .,\mu_{20}\}$$ (18)

#### 2.2.7. Determination of reverse accumulative absolute position incidence vector (RAAPIV)

Similarly, 20 elements of reverse accumulated absolute position incidence vector (RAAPIV) is formed (as shown in Eq (20)) by simply reversing the primary structure of the *p*^*th*^ residue (as shown in Eq (19)).

$$\alpha_{pn}^{i},\;\ldots ,\;\alpha_{p(n-1)}^{i},\;\ldots ,\;\alpha_{p1}^{i}$$ (19)

$$RAAPIV=\{{\mu^{\prime }}_{1}.{\mu^{\prime }}_{2},\ldots .,{\mu^{\prime }}_{20}\}$$ (20)

### 2.3. Prediction model

**Fig 2:**
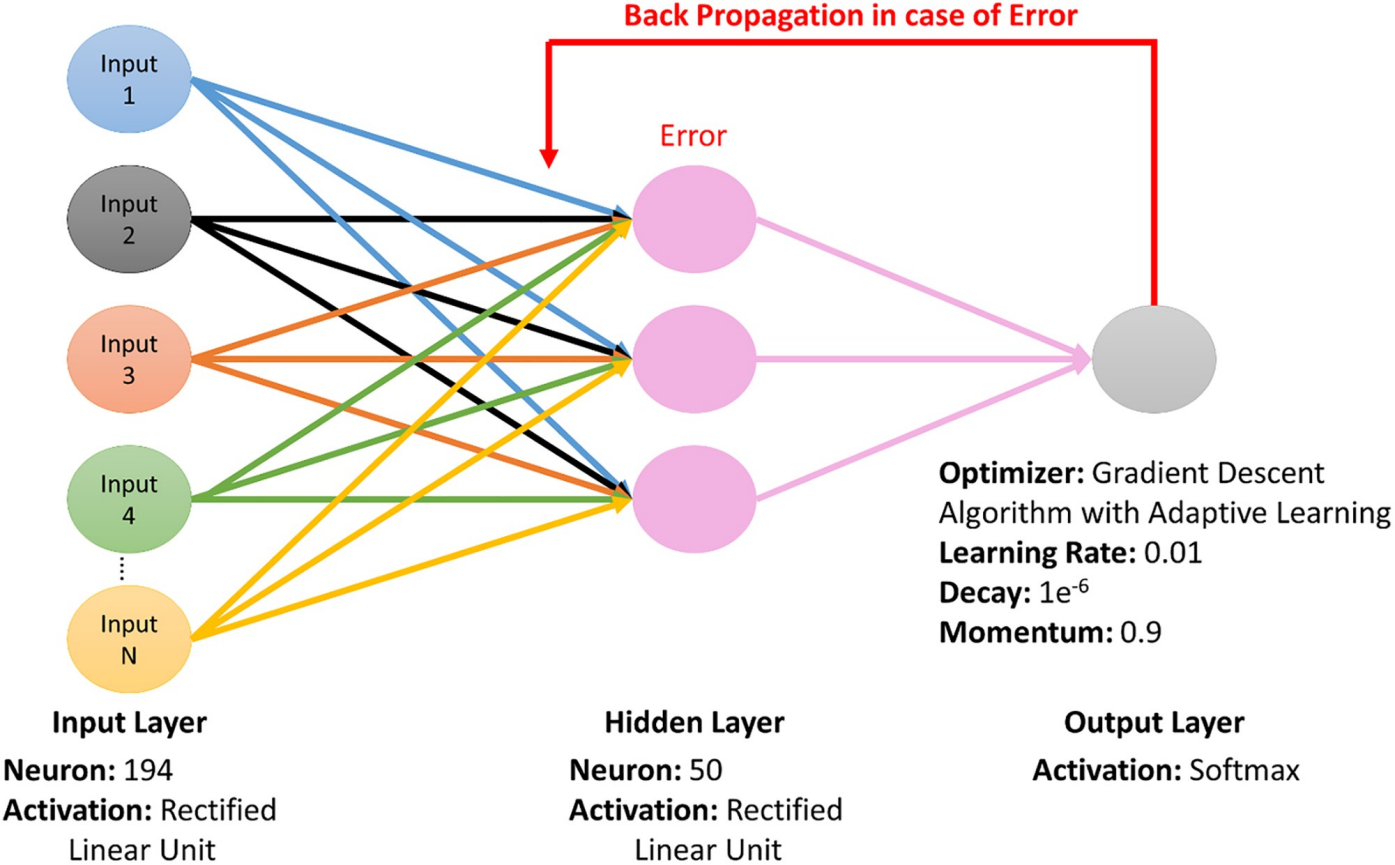
The architecture of ANN for the proposed prediction model.

Billions of neurons are present in the human brain, which works by taking in the information, processing it and then passing further for its functioning. Each time, patterns are being learned and information is extracted which is used to act against situations and things with no specificity. ANN (Artificial Neural Network) is a system, based on technique like the human brain. Information is being learned from various situations and patterns. Later on, it uses interconnect neurons to works in problem-solving. A number of neurons take the input and previous information, along with patterns are used. There are two working modes of ANN, the first one is the training mode, in which ANN is being trained on provided data and information is extracted from that set of data and learn. The second one is using or working, it’s on working mode; like neurons provide input and best match for output is extracted using available information [68, 69] and taught patterns as shown in Fig 2.

For this prediction model, ANN is implemented with back propagation methods, to reduce the error. For the specific and standard dataset, feature extraction was conducted and feature vector consisted of central, Hahn, and raw moments of sequence matrix, RPRIM and PRIM; FV, SVV, RAAPIV and AAPIV. The information linked to the position of proteins and representation was protected and saved in the form of final Feature Vector (FV) and these were total of 194. IFM (Input Feature Matrix) was made using all these FV and each row in IFM shows the FV related to every sample of the specific benchmark dataset. For each sample output, OM (the Output Matrix) was produced using all these output labels of input from IFM. ANN was being trained using both OM and IFM, OM to reduce the error by back propagation method and IFM as input [70, 71] as shown in Fig 2.

The neural Network was trained and tested using the Python version 3.4 along with web application framework Flask.

## 3. Results and discussion

### 3.1. Estimated accuracy

During the production of any new model, objective evaluation of its rate is one of the most important objectives. In order to justify the evaluation of the model, two questions must be catered. 1) What type of metrics should we use to review the quality of prediction model? 2) What are the various test methods that can be used for score metrics?

### 3.2. Formulation of metrics

Following are the four different evaluation metrics, used to evaluate the precision of the prediction model. (1) Acc to estimate the prediction model’s overall accuracy, (2) *S*_*n*_ for the sensitivity of the prediction model, (3) *S*_*p*_ for the specificity of the prediction model, (4) MCC for the stability of the prediction model to access the accuracy and quality of the prediction model, various conventional metrics which have been used frequently in literature, are no more beneficial, because of lack in insightfulness and difficulty faced by the biologist in understanding it. Particularly for the Mathew’s Correlation Coefficient (MCC), which is highly significant in illustrating the stability of the prediction model. Conveniently, various symbols have been an introduction to study protein signal peptides by Chou [72], A set of four intuitive equation were derived [13, 21, 73] as follows in Eq (21).

$$\{\begin{array}{ll} Sensitivity\;\left(S_{n}\right)=1-\frac{N_{-}^{+}}{N^{+}} & 0\;\leq S_{n}\leq 1\\ Specificity\;\left(S_{p}\right)=1-\frac{N_{+}^{-}}{N^{-}} & 0\leq S_{p}\leq 1\\ Accuracy\;\left(Acc\right)=1-\frac{N_{-}^{+}+N_{+}^{-}}{N^{+}+N^{-}} & 0\leq Acc\leq 1\\ MCC=\frac{1-\left(\frac{N_{-}^{+}}{N^{+}}+\frac{N_{+}^{-}}{N^{-}}\right)}{\sqrt{\left(1+\frac{N_{+}^{-}-N_{-}^{+}}{N^{+}}\right)\;\left(1+\frac{N_{-}^{+}-N_{+}^{-}}{N^{-}}\right)}} & -1\leq MCC\leq 1 \end{array}$$ (21)

$N_{+}^{-}$ illustrates the number of the incorrectly predicted non-crotonyllysine sites as crotonyllysine sites. N^−^ represents the number of the correctly predicted non- crotonyllysine sites. Furthermore, N^+^ presents the number of the correctly predicted crotonyllysine sites. $N_{-}^{+}$ represents the number of the incorrectly predicted crotonyllysine sites as non- crotonyllysine sites.

From Eq (21), it can be seen that when $N_{-}^{+}=0$ it means that S_*n*_ = 1, because not a single crotonyllysine site is predicted as the non- crotonyllysine sites. If $N_{-}^{+}=N^{+}$, then S_*n*_ = 0, as all the crotonyllysine sites are incorrectly predicted as non- crotonyllysine sites. Additionally, if $N_{+}^{-}=0$, then the specificity is Sp = 1, which means none of the single non- crotonyllysine site is incorrectly predicted as a crotonyllysine site; while if we have $N_{+}^{-}=N^{-}$, then the specificity is Sp = 0 as all the non- crotonyllysine sites. If $N_{-}^{+}=N_{+}^{-}=0$, it shows that not a single non- crotonyllysine site in the negative dataset and crotonyllysine site in the positive dataset incorrectly predicted, and it provides us with the Acc = 1 and MCC = 1; if we have $N_{+}^{-}=N^{-}$, and $N_{-}^{+}=N^{+}$, it shows that all the non-crotonyllysine sites in the negative dataset and all the crotonyllysine sites in the positive dataset are incorrectly predicted, and it provides us with the Acc = 0 and MCC = −1. Whereas, if $N_{-}^{+}=N^{+}/2$ and $N_{+}^{-}=N^{-}/2$ then it will provide us with the Acc = 0.5 and MCC = 0, leaving us with doubt, whether it is non- crotonyllysine site or crotonyllysine site. Overall, Eq (21) illustrates the explanation of sensitivity, specificity, overall accuracy in relation to MCC [74, 75].

These perceptive metrics have been adapted and reported by various modern publications (see, e.g. [76–79]. In Eq (21) the set of defined notations can only successfully function for binary labelled data, like whether the predicting site is crotonyllysine or non- crotonyllysine. In case of multi-label prediction, the problem is completely different, which becomes more general in biomedicine [80] and computational biology [81], so required a different type of metrics [82].

### 3.3. Self-consistency testing

On iCrotoK-PseAAC, self-consistency test was implemented which is to use the same benchmark dataset to first train and then test the proposed model [73]. When true positive (TP) value is already familiar, then such a data is generally used. In the following Table 1, results are shown, which provides the predicted and actual classification compiled by the proposed computational model. It describes the overall presentation of the proposed systems.

**Table 1:** Self-consistency testing results for iCrotoK-PseAAC.

Predictor	Accuracy Metrics			
	Accuracy (%)	Specificity (%)	Sensitivity (%)	MCC
**iCrotoK-PseAAC**	100	100	100	1

### 3.4. Testing via 10-fold cross-validation

Authentic datasets are not available always for validation of model, thus, in that case, cross-validation is chosen to develop the exception that the proposed model is predicting accurately [73].

During the cross-validation, k is considered as constant when the disjoint k-fold dataset is achieved after breaking up. Testing is executed k-times for each and every partition after training and accuracy is computed for each of the reiterations. In the end, the average or mean of all the accuracies is recorded as cross-validation. For both positive and negative data samples, the same technique was correlated. To make the subsets for *k* = 10, a casual selection was conducted as compared to the other methods of validation. Table 2 reports the 10-fold cross-validations results for iCrotoK-PseAAC.

**Table 2:** 10-fold cross-validation results for iCrotoK-PseAAC (average of 10 folds).

Predictor	Accuracy Metrics			
	Accuracy (%)	Specificity (%)	Sensitivity (%)	MCC
**iCrotoK-PseAAC**	99.17	99.53	99.40	0.98

### 3.5. Comparative analysis

Different techniques have reported different results but our methodology has improved the accuracy, specificity but reduced the sensitivity and also improved the Mathew’s Co-relation Coefficient. The results were compared with iKcr-PseEns [10] and CKSAAP_CrotSite [9] predictor, the most recent method for predicting crotonyllysine sites. In Table 3 the results of all the four matrices *Sn*, *Sp*, *Acc*, and *MCC* are depicted, signifying that anticipated predictor. It is observed that the proposed predictor performs better in terms of accuracy, specificity, and sensitivity as compared to the previously reported methods using independent data set.

**Table 3:** Comparative analysis of methods.

Predictor	Accuracy Metrics			
	Accuracy (%)	Specificity (%)	Sensitivity (%)	MCC
**iKcr-PseEns** [10]	94.49	95.27	90.53	0.81
**CKSAAP_CrotSite** [9]	98.11	99.17	92.45	0.9283
**iCrotoK-PseAAC**	99.17	99.53	99.40	0.98

## 4. Webserver

Development of a webserver which is user-friendly; is the last 5-step rule. Publicly accessible and user-friendly webserver provides the future directions for making prediction methods and computational tools which will be more useful practically as demonstrated in the recent publications [13, 19, 21, 22, 73, 83, 84]. Specifically, these useful and practical webservers have an increasing effect on medical sciences, leading medicinal chemistry into an unequalled revolution [48], thus, efforts will be made to construct a webserver for the prediction model reported in this paper.

## 5. Conclusion

Among different post-translational modifications (PTMs), one of the most important ones is lysine crotonylation in proteins. The initial and crucial step is to identify crotonylation occurrence in protein along with their sites to fully understand the mechanism behind these biological processes. The rigid and imbalanced nature of dataset of protein-peptide makes it difficult to understand and time-consuming which affects the precision of the prediction model. We weren’t able to get efficient results of sensitivity yet, like accuracy, specificity and MCC. So, the essential demands of computational methods to predict the sites of Crotonylation are highly justified. Our proposed model, iCrotoK-PseAAC which used statistical moments and position relativity to increase the accuracy of the site prediction of lysine Crotonylation. Our predictor model has used SVV, SM, FV, PRIM, RPRIM, AAPIV and RAAPIV (as we discussed above) to compute the accuracy, sensitivity, specificity and MCC. The results of independent dataset testing were 99% accuracy, 89.1% sensitivity, 99.4% specificity and 0.98 MCC. Since our major emphasis was on increasing the accuracy of sites prediction of lysine Crotonylation, which we have achieved.

## Supporting information

S1 File Dataset containing 378 positive sample and 500 negative samples. (XLSX)
